# Draft Genome Sequence of Venenivibrio stagnispumantis CP.B2^T^, Isolated from Champagne Pool, Waiotapu, Aotearoa-New Zealand

**DOI:** 10.1128/mra.01074-22

**Published:** 2023-01-25

**Authors:** Jean F. Power, Holly E. Welford, Carlo R. Carere, Ian R. McDonald, S. Craig Cary, Matthew B. Stott

**Affiliations:** a Thermophile Research Unit, Te Aka Mātuatua-School of Science, Te Whare Wānanga o Waikato, University of Waikato, Hamilton, Aotearoa-New Zealand; b Te Kura Pūtaiao Koiora-School of Biological Sciences, Te Whare Wānanga o Waitaha, University of Canterbury, Christchurch, Aotearoa-New Zealand; c Te Tari Pūhanga Tukanga Matū-Department of Chemical and Process Engineering, Te Whare Wānanga o Waitaha, University of Canterbury, Christchurch, Aotearoa-New Zealand; d Biomolecular Interaction Centre, Te Whare Wānanga o Waitaha, University of Canterbury, Christchurch, Aotearoa-New Zealand; University of Southern California

## Abstract

Venenivibrio stagnispumantis strain CP.B2^T^ is a thermophilic, chemolithoautotrophic bacterium from the family *Hydrogenothermaceae* (phylum *Aquificota*), isolated from Champagne Pool in the Waiotapu geothermal field, Aotearoa-New Zealand. The genome consists of 1.73 Mbp in 451 contigs with a 30.8 mol% G+C content.

## ANNOUNCEMENT

The bacterial genus *Venenivibrio,* within the phylum *Aquificota* (family *Hydrogenothermaceae*), is characterized by thermophilic microaerophilic hydrogenotrophs (1–3). The type strain for the genus, Venenivibrio stagnispumantis CP.B2^T^, was isolated from Champagne Pool, Waiotapu, Aotearoa-New Zealand, a geothermal system under the *kaitiakitanga* (guardianship) of Māori *iwi* (tribe) Ngāti Tahu-Ngāti Whaoa. The V. stagnispumantis genome has previously been sequenced as part of the Genomic Encyclopedia of Bacteria and Archaea (GEBA) project KMG-II (4) (IMG genome ID 2724679818). However, we have resequenced the genome on behalf of Ngāti Tahu-Ngāti Whaoa to recognize indigenous data sovereignty and to assert *mana whenua* (customary rights) over the bacterium, its genome, and the location in which it was isolated.

Venenivibrio stagnispumantis CP.B2^T^ (obtained from the University of Waikato, Aotearoa-New Zealand) was cultivated using a modified MSH medium (70°C, pH 5.5) within a headspace consisting of N_2_/H_2_/CO_2_/O_2_ at ratios of 50:40:7.5:2.5 (vol/vol), respectively (1). A total DNA extract (3.5 ng μl^−1^) of CP.B2^T^ was performed using a NucleoSpin Soil kit (Macherey-Nagel, Düren, Germany) according to the manufacturer’s protocols. Whole-genome sequencing (Macrogen, Inc., Seoul, South Korea) was undertaken using the Illumina HiSeq 2500 platform with TruSeq DNA Nano (550) library preparation for paired-end 101-bp reads (Illumina, Inc., San Diego, CA, USA). Default parameters were used for all assembly, quality control (QC), and annotation software unless otherwise specified. A total of 3.8-Gbp raw sequences (37.32 million reads) were assembled using SPAdes v3.12.0 (5) with the “–careful” option to minimize mismatches and kmer sizes of 21, 33, and 55. The assembly was evaluated using QUAST v5.0.0 (6). The genome was annotated via the NCBI Prokaryotic Genome Annotation Pipeline (PGAP; v6.3 [7]) (GenBank accession no. JAPEIW010000000) using the GeneMarkS-2+ protein reference set. A functionally equivalent annotation of 47 scaffolds using the Integrated Microbial Genomes annotation pipeline v4.16.4 (8) is also available (IMG genome ID 2799112217).

The V. stagnispumantis CP.B2^T^ genome comprised 1,731,156 bp containing 2,034 protein-coding genes, with 1,963 having predicted function. The genome consisted of 451 contigs, with a total G+C content of 30.8% mol. The *N*_50_ value was 91,033, the *L*_50_ was 8, and genome coverage was ~2,242×. The genome encodes 49 RNAs, including single complete copies of the 5S, 16S, and 23S rRNAs; 36 tRNAs; and 3 noncoding RNAs (ncRNAs).

A pairwise average nucleotide identity (ANI) comparison using FastANI v0.1.3 (9) of the V. stagnispumantis CP.B2^T^, Sulfurihydrogenibium yellowstonense SS-5^T^, and Persephonella marina EX-H1^T^ genomes gave <80% similarity for each, confirming the designation of *Venenivibrio* as a separate genus to sister genera *Sulfurihydrogenibium* and *Persephonella*.

### Data availability.

Raw sequences for the V. stagnispumantis CP.B2^T^ genome were deposited in the European Nucleotide Archive under the BioProject accession no. PRJEB55610. The assembled genome was deposited in the Genomes Online Database (10) (GOLD analysis ID Ga0591133), for associated annotation with the Integrated Microbial Genomes & Microbiomes platform (8) (IMG genome ID 2799112217). This Whole Genome Shotgun project has been deposited at DDBJ/ENA/GenBank under accession no. JAPEIW010000000. The version described in this paper is version JAPEIW010000000.1.
